# LCWG-DETR: a wavelet-enhanced detection transformer for citrus disease detection improving visual perception in harvesting

**DOI:** 10.3389/fpls.2026.1942627

**Published:** 2026-09-09

**Authors:** Dalin Zhang, Baijing Wu, Ke Gao, Guanghui Yan

**Affiliations:** 1 College of Information Technology, Nanchang Vocational University, Jiangxi, China; 2 School of Electronic and Information Engineering, Lanzhou Jiaotong University, Lanzhou, China

**Keywords:** citrus disease detection, citrus harvesting robot, directional attention, multiscale feature fusion, visual perception system

## Abstract

To address the low detection accuracy and frequent missed detections of citrus diseases caused by leaf occlusion, small lesion sizes, and high visual similarity among disease categories in complex orchard environments, this study proposes LCWG-DETR, a citrus disease detection method that combines wavelet edge features with Gaussian distance regression. First, an LGFE-ResNet18 network is developed for feature extraction. It integrates texture perception guided by the standard deviation of local intensity deviations with global structural encoding in the Fourier frequency domain. This design compensates for the attenuation of high frequency information from small lesions caused by stacked convolutional layers and improves the extraction of subtle disease features. Next, a cross scale adaptive feature fusion module, termed CAFF, is introduced. It employs progressive layerwise fusion and dynamic weighting to reduce the loss of spatial details during upsampling. In addition, a wavelet directional attention module, termed WDAM, is constructed. Haar wavelet decomposition is used to extract horizontal and vertical high frequency components and generate directional modulation weights, thereby guiding the network to focus more accurately on lesion boundaries and regions with abrupt texture variations. Finally, a Gaussian composite distance loss, termed GCD, is introduced. It represents bounding boxes as two dimensional Gaussian distributions with covariance matrices, which alleviates vanishing gradients and scale sensitivity during small lesion regression. Experimental results on the self collected JXDF dataset and the public OFDD dataset show that LCWG-DETR improves mAP by 5.07% and 3.41%, respectively, compared with the baseline model, while reducing the number of parameters by 0.35 M. These results demonstrate that the proposed method achieves a favorable balance between detection accuracy and real time performance. Moreover, it maintains stable detection performance and strong generalization under typical conditions involving occlusion, dense targets, backlighting, and complex backgrounds, providing reliable technical support for the visual perception systems of citrus harvesting robots.

## 1 Introduction

Citrus is one of the most widely cultivated and extensively traded fruit crops worldwide. However, its production and fruit quality are severely threatened by various diseases, including citrus canker, Huanglongbing, black spot, and anthracnose. These diseases can cause premature fruit drop, fruit decay, and tree decline, while also reducing the commercial grade and processing suitability of harvested fruit. Consequently, disease has become one of the primary biological constraints on the sustainable development of the citrus industry (Yu et al., 2025). According to data from the Food and Agriculture Organization of the United Nations (Singh et al., 2026), more than 20% of global citrus fruit biomass is lost during preharvest and postharvest stages because of diseases and other biological stresses. In some major production regions, the spread of destructive diseases such as Huanglongbing has resulted in even more severe yield losses. Citrus fruit diseases are commonly characterized by visible symptoms on the peel, including black spots, canker lesions, brown necrotic areas, and abnormal coloration. Therefore, rapid and accurate disease identification from fruit appearance images can support the precise removal of infected fruit during harvesting and grading, thereby reducing postharvest decay, increasing the proportion of marketable fruit, and improving competitiveness in export markets. However, conventional manual sorting and field inspection are labor intensive, subjective, and often delayed, making them unsuitable for the early detection and precise postharvest grading required in large scale citrus production. Therefore, the development of efficient, objective, and automated methods for citrus fruit disease detection has become an urgent requirement for ensuring the safety and sustainable development of the citrus industry (Sharma and Abrol, 2025). The proposed workflow first employs a deep learning based detection algorithm to identify healthy citrus fruits under occlusion and dense small target conditions, obtaining their confidence scores, category labels, and bounding box locations. The detected results are then combined with the intrinsic camera parameters to estimate the three dimensional spatial coordinates of the fruits. Finally, these three dimensional coordinates are used as inputs to the path planning algorithm to determine the joint angles of the harvesting manipulator and generate an optimal collision free motion trajectory.

In recent years, the rapid development of next generation information technologies, including the Internet of Things, big data, and artificial intelligence, has provided essential technical support for the digital and intelligent transformation of agriculture. In this context, artificial intelligence has become a key technology for supporting refined management and scientific decision making throughout the agricultural production cycle because of its strong capabilities in image recognition, data mining, and predictive modeling (Guo et al., 2025). Owing to its well established industrial chain, high economic value, and urgent demand for technological innovation, the citrus industry represents a suitable application scenario for the integration and demonstration of smart agricultural technologies. Within intelligent citrus production systems, efficient and accurate disease detection is essential for maintaining crop health and improving production efficiency. Conventional manual inspection is limited by low efficiency, strong subjectivity, and delayed responses, making it difficult to meet the requirements of large scale orchards for early disease identification and precise disease control. Deep learning methods for image based disease detection train convolutional neural networks and related models to analyze citrus leaf images and identify disease symptoms in real time. These methods provide a reliable foundation for intelligent and nondestructive monitoring of citrus diseases (Wu et al., 2026).

Although considerable progress has been made in citrus disease detection, existing methods still face substantial challenges in complex cultivation environments (Mai et al., 2026). Many models are trained on datasets collected under controlled conditions with relatively simple backgrounds. Consequently, they often struggle to accommodate variations in illumination, occlusion by branches and leaves, and diverse disease appearances in real orchard scenes, resulting in limited robustness and generalization. In addition, the acquisition and annotation of disease images under field conditions are costly and labor intensive, while large scale, high quality public datasets remain scarce. These limitations restrict further improvements in model performance and hinder practical deployment. To address these challenges, citrus disease detection methods have gradually evolved from traditional machine learning approaches to deep learning models. Conventional machine learning methods generally rely on manually designed features, such as color and texture descriptors, combined with traditional classifiers to identify disease symptoms. However, their limited feature representation capacity often leads to poor generalization in complex orchard environments. With the rapid development of deep learning, convolutional neural networks have been increasingly used to automatically learn discriminative disease features. This approach reduces the dependence on handcrafted feature design and improves both detection accuracy and robustness (Injante and Sánchez Isuiza, 2025). On this basis, the introduction of object detection frameworks and lightweight network designs has further promoted the deployment of disease detection models in practical applications (Luo et al., 2025). Nevertheless, most existing studies remain focused on specific disease categories, controlled environments, or idealized datasets. Under real orchard conditions, citrus fruits are often densely distributed and partially occluded, while complex backgrounds introduce substantial visual interference. Moreover, disease lesions are frequently small, irregular, and unevenly distributed. These factors continue to limit the ability of current models to generalize across different scenes and accurately detect occluded or subtle disease symptoms. Therefore, achieving accurate and robust citrus fruit disease detection under complex cultivation conditions remains an important challenge that requires further investigation.

To address the above challenges, this study develops LCWG-DETR (Detection Transformer with LGFE-ResNet18, CAFF, WDAM, and GCD), a visual perception method for citrus harvesting robots that integrates wavelet edge features with Gaussian distance regression. The proposed framework is improved from four aspects, including feature extraction, cross scale feature fusion, deep feature enhancement, and bounding box regression. These components form a complete optimization pipeline that extends from subtle lesion perception to precise target localization. The main contributions are summarized as follows.

1. Local-Global Feature Enhancement ResNet-18 (LGFE-ResNet18). The LGFE module is embedded into the C2f structure of ResNet-18 to construct a dual-branch architecture consisting of local deviation texture feature extraction and global Fourier frequency-domain feature extraction. By jointly modeling the frequency-domain responses and spatial textures of subtle early-stage lesions during feature extraction, LGFE-ResNet18 effectively compensates for the attenuation of high-frequency information from small targets caused by stacked convolution operations, thereby improving the sensitivity and discriminative capability of the network for weak lesion features.
2. Cross-Scale Adaptive Feature Fusion (CAFF). A Weighted Feature Fusion (WFF) module is designed to enable dynamic interaction between shallow high-resolution details and deep semantic information. Combined with the progressive enhancement of RepC3 and an adaptive weighting mechanism, CAFF strengthens feature responses in occluded regions while injecting shallow spatial details into deeper features. This design effectively compensates for spatial information loss caused by upsampling and alleviates blurred lesion boundaries and localization deviations under occlusion.
3. Wavelet-based Directional Attention Module (WDAM). The Haar discrete wavelet transform is employed to extract horizontal and vertical high-frequency components from feature maps and generate directional modulation weights. These weights guide the attention mechanism through elementwise modulation to preferentially respond to lesion boundaries and regions with abrupt texture variations. In this way, WDAM suppresses interference from complex orchard backgrounds while maintaining a relatively low computational cost.
4. Gaussian Combined Distance loss (GCD). Bounding boxes are modeled as two-dimensional Gaussian distributions, and their center distances and covariance matrices are jointly optimized. This formulation provides stronger gradient responses for extremely small lesions, effectively alleviating gradient attenuation during small-target regression and enabling more precise localization and accurate regression of disease bounding boxes.

## 2 Related works

Faced with major challenges such as continuous global population growth, increasingly limited arable land resources, and intensifying climate change, the limitations of traditional agricultural management, including its reliance on experience based decision making and insufficient support from scientific data, have become increasingly evident. Intelligent technologies are therefore urgently needed to transform conventional agricultural production. Artificial Intelligence (AI) has emerged as a major driving force of the fourth agricultural revolution and has created new opportunities for modern agricultural production. Through data driven approaches, AI technologies have demonstrated significant advantages in key applications such as crop growth monitoring, disaster early warning, water and fertilizer management, and automated agricultural operations, providing important technical support for addressing these challenges (Muhammad et al., 2026). Following this technological trend, researchers worldwide have conducted extensive studies on crop remote sensing monitoring, pest and disease recognition, precision irrigation, and agricultural robotics. As an important application of smart agriculture in horticultural production, citrus disease detection has evolved closely with the broader development of AI. Accordingly, related studies are reviewed below from two perspectives: general AI applications in agriculture and specific citrus disease detection tasks.

Macro Applications of Artificial Intelligence in Smart Agriculture. In crop remote sensing and yield prediction (Radočaj et al., 2026), systematically reviewed satellite remote sensing based crop yield prediction studies published between 2000 and 2025, covering the application of multispectral data from Sentinel-2, Landsat, and MODIS to agricultural yield estimation. Their review showed that this field has gradually evolved from simple regression toward machine learning, deep learning, and multisource data fusion frameworks. Although the study provides a comprehensive overview of technological development, it does not sufficiently discuss limitations related to the interpretability of deep learning models or their weak integration with physical mechanisms (Yang et al., 2025). integrated unmanned aerial vehicle (UAV) RGB and multispectral remote sensing data with algorithms such as Random Forest (RF) to construct chlorophyll content inversion and yield prediction models, enabling high throughput phenotypic monitoring and accurate prediction of cotton varieties. However, the performance of these models depends strongly on the temporal quality of remote sensing data acquired at specific growth stages, which may limit their practical application (Chen et al., 2025). assimilated remote sensing observations into the Decision Support System for Agrotechnology Transfer (DSSAT) crop model using a Markov Chain Monte Carlo (MCMC) method, effectively alleviating the saturation problem of the Normalized Difference Vegetation Index (NDVI). Although this approach provides strong physical interpretability, its high computational complexity makes it difficult to satisfy the response requirements of real time field management (Khan et al., 2025). developed a Multi Stream Deep Neural Network (MSDNN) for county level maize yield estimation using the Normalized Difference Vegetation Index (NDVI) and Enhanced Vegetation Index (EVI) derived from MODIS data. However, the moderate to low spatial resolution of MODIS limits its applicability to fine scale field management and smallholder farming systems. In pest and disease recognition and precision crop protection (Zhao et al., 2023), systematically reviewed the mechanisms and applications of low altitude unmanned aerial vehicle (UAV) remote sensing for crop pest and disease monitoring, providing technical references for prescription map based precision spraying. However, their work mainly focuses on large scale monitoring and gives limited attention to fine grained disease recognition and severity assessment at the individual plant level (Guan et al., 2026). proposed the PCSNet detection model based on YOLOv12, which combines Coordinate Attention (CA) with a shallow feature enhancement branch to detect densely distributed small winged aphids on sticky traps. Although this method provides an effective solution for intelligent pest monitoring, the additional attention mechanism increases computational cost and therefore still requires further lightweight optimization for deployment on edge devices (Wu et al., 2026). proposed DBG-DETR, which reduces model complexity through Dynamic Multiscale Gated Fusion and a Gated Sparse Dynamic Transformer. However, the dynamic gating strategy relies on predefined thresholds, leaving room for further improvement in adaptability across different disease types and growing seasons. In precision irrigation and agricultural robotics (Bilal et al., 2025), developed a Takagi-Sugeno based fault detection framework for quadrotor unmanned aerial vehicles, although its robustness under highly variable agricultural weather conditions has not yet been sufficiently validated (Dobre et al., 2024). demonstrated the practical value of the Internet of Things (IoT) in smart agriculture using the Libelium IoT platform as an example, but provided limited analysis of data security and long term operation and maintenance costs (Dai et al., 2024). proposed a Multi Objective Discrete Artificial Bee Colony algorithm for task allocation in harvesting robots. However, its practical scheduling performance under signal occlusion and localization drift in unstructured agricultural environments still requires further verification.

Technical Evolution of Citrus Disease Detection and Recognition Methods. Citrus yield and quality are seriously threatened by destructive diseases such as Huanglongbing, citrus canker, and black spot, making accurate identification and targeted disease control critical to the sustainable development of the citrus industry (Khamsaw et al., 2022; Krajewski et al., 2022). To address this problem, research has gradually evolved from traditional machine learning methods to deep learning based approaches. Traditional methods generally follow a pipeline consisting of manually designed features and shallow classifiers (Mai et al., 2026) (Ali et al., 2017). used the Delta E color difference algorithm to segment lesion regions, extracted color and texture features, and then applied Principal Component Analysis (PCA) for dimensionality reduction before classification. Although this method established a complete disease recognition pipeline, it was sensitive to illumination variations and showed limited generalization ability (Sankaran et al., 2010). reviewed three categories of ground based sensing technologies, including spectroscopy, imaging, and volatile compound sensing, providing useful guidance for sensor selection, but giving limited attention to multisource data fusion strategies (Barman et al., 2023). used 13 color and texture features together with a polynomial kernel Support Vector Machine (SVM) to achieve early disease classification. This method requires relatively low computational resources, but its performance depends strongly on kernel selection and shows limited generalization across citrus varieties. Convolutional Neural Network (CNN) based methods automatically learn hierarchical representations through stacked convolution and pooling layers, reducing the dependence on manually designed features (Mir et al., 2025). employed DenseNet121 for four class citrus disease classification and achieved an accuracy of 96.14%. However, the experiments were conducted using images collected under laboratory conditions, and the performance degradation in real orchard environments was not sufficiently evaluated (Goyal and Lakhwani, 2025). compared four CNN models on a nine class dataset, providing a useful benchmark, although the dataset contained only 759 images (Dhiman et al., 2023). combined CNN and Long Short Term Memory (LSTM) networks and further employed pruning and quantization for edge deployment. Although this work provided a useful exploration of model compression, the influence of compression induced accuracy loss on fine grained disease classification was not quantitatively analyzed. Nevertheless, CNN based methods are inherently constrained by fixed receptive fields, which limits their ability to model global context and long range dependencies. Transformer based methods address this limitation through the self-attention mechanism (Karthik et al., 2023). proposed a dual branch deep fusion framework that combines local CNN features with global contextual information extracted by the Swin Transformer and further enhances the fused representation using Shuffle Attention. Although this design achieves complementary local and global feature modeling, the dual branch architecture introduces a substantial increase in model parameters (Gao et al., 2026). proposed a lightweight LBM detection Transformer that reduces computational cost through large kernel attention and multiscale linear attention, but its localization accuracy for densely distributed small targets still requires improvement (Eman et al., 2025). employed MobileNetV2 and Bidirectional Encoder Representation from Image Transformers (BEiT) as dual feature extractors and used a Support Vector Machine (SVM) for classification after feature fusion. This approach combines computational efficiency with global modeling capability, but the use of SVM limits the advantages of end to end training (Liu et al., 2025). improved RT-DETR and proposed MSHLB-DETR, in which the SDRM module strengthens local and global feature interaction. However, its sensitivity to subtle mottling patterns associated with early Huanglongbing remains limited, and further reduction in model complexity is still required.

Overall, existing methods still face several challenges in real orchard environments. Occlusion, illumination variation, and large differences in disease scale make small disease target detection particularly difficult. Meanwhile, many methods rely on increasingly complex network structures to improve detection accuracy, resulting in large parameter counts and high inference latency, which makes it difficult to satisfy the real time and lightweight requirements of edge platforms. In addition, most existing studies treat disease recognition as an independent task and do not integrate it into the complete perception, decision, and execution workflow of harvesting robots. To address these limitations, this study focuses on practical citrus harvesting scenarios and introduces targeted improvements in four key stages: feature extraction, cross scale feature fusion, attention enhancement, and bounding box regression. Lightweight design is incorporated as an explicit constraint throughout the framework to reduce computational cost while maintaining high detection accuracy. In this way, the proposed method provides an accurate and real time visual perception solution for citrus harvesting robots and establishes a reliable basis for subsequent three dimensional localization and motion planning.

## 3 LCWG-DETR

The baseline RT-DETR model (Zhao et al., 2024) mainly consists of five components: a ResNet-18 feature extraction backbone, AIFI(an Adaptive Intra Scale Feature Interaction module), CCFM(a Cross Scale Feature Fusion Module), an uncertainty minimal query selection, and a detection head. However, several limitations arise when this framework is applied to complex citrus orchard scenes characterized by severe occlusion and small, visually similar lesions. In ResNet-18, repeated convolution and pooling operations limit the ability of shallow layers to capture subtle texture variations associated with early disease symptoms. Meanwhile, progressive downsampling continuously reduces spatial resolution, causing the positional information of small lesions to become substantially weakened in deep feature maps and resulting in insufficient feature representation. AIFI performs self-attention interaction only on the feature map with the lowest spatial resolution. At this stage, the semantic responses of small lesions are often weak, whereas large healthy regions and prominent leaf veins dominate the global attention distribution. Consequently, disease related features from small targets may be overlooked. During cross scale feature fusion, CCFM cannot recover spatial details that have already been lost through downsampling. Moreover, convolution based fusion may combine noise from occluded regions with genuine lesion features. The absence of a selective attention mechanism further leads to localization deviations and reduced confidence for partially occluded lesions. Finally, because small lesions generally produce weak responses, low confidence scores, and high localization uncertainty, they are less likely to be selected by the uncertainty minimal query selection module as initial object queries. As a result, some small lesions may be excluded before decoding and remain undetected.

**Figure 1:**
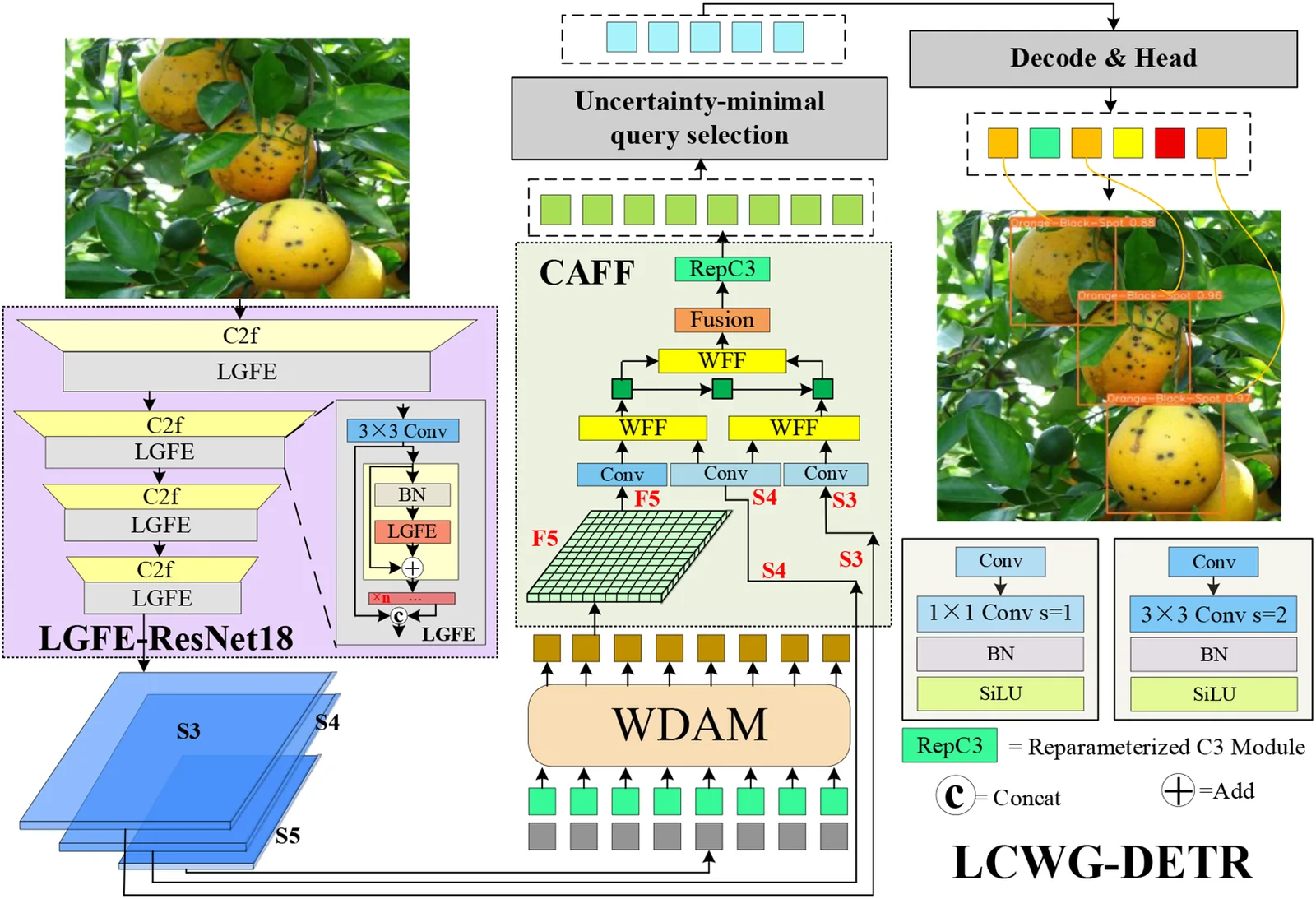
Network architecture of LCWG-DETR.

To address the low detection accuracy and frequent missed detections of citrus diseases caused by dense leaf occlusion, extremely small lesion scales, and high interclass visual similarity in complex orchard environments, this study proposes the LCWG-DETR detection framework, whose overall architecture is shown in **Figure 1**. The proposed method adopts ResNet18 embedded with LGFE as the backbone network. In the four C2f stages of ResNet18, wavelet transform is introduced to decompose feature maps into low frequency approximation components and high frequency detail components. By exploiting the strong responses of high frequency components to citrus disease edge textures and fine lesion boundaries, the network enhances its ability to represent disease features in low resolution and occluded regions, thereby effectively alleviating the suppression of high frequency information from tiny lesions caused by standard convolution based downsampling operations. Subsequently, the multiscale feature maps S3, S4, and S5 from the last three stages of the backbone are selected. Among them, S5 has the largest receptive field and contains the richest global semantic information, whereas S3 and S4 retain higher spatial resolution and are therefore more suitable for small target discrimination. In the feature enhancement stage, S5 is fed into WDAM. This module evaluates the disease response intensity at each spatial location through dynamic gating and selects the Top-K positions with the strongest discriminative responses for attention weighted aggregation according to their response scores. In this way, the network focuses on weak response regions associated with small and occluded disease targets within the global context, thereby activating and strengthening discriminative features and producing the enhanced feature F5. In the cross scale fusion stage, F5 together with S3 and S4, is fed into CAFF. The module constructs a bidirectionally densely connected multiscale pyramid interaction structure. High level semantic information is propagated from top to bottom to enhance the discriminative capability of low level features, while spatial details from lower level features compensate for the resolution degradation of high level representations. Meanwhile, dense cross layer connections facilitate information exchange among different feature levels, effectively reducing information dilution and detail loss during multiscale fusion. This design improves the robustness of the model in discriminating and localizing disease regions under complex orchard conditions involving occlusion, illumination variation, and diverse lesion morphologies. Finally, the fused multiscale features are processed by an uncertainty minimal query selection strategy to generate high quality initial object queries, which are then fed into the detection head for category classification and bounding box regression. GCD is further introduced to constrain the distribution similarity between the predicted and ground truth bounding boxes. The final citrus disease detection results are then obtained, providing accurate disease perception support for the visual system of citrus harvesting robots.

### 3.1 LGFE-ResNet18

In ResNet-18, stacked convolution and pooling operations restrict the receptive field of shallow layers, making it difficult to capture subtle lesion texture patterns. Meanwhile, progressive downsampling continuously reduces spatial resolution, causing the positional information of small lesions to be gradually weakened in deeper feature maps and ultimately resulting in insufficient feature extraction. To address these limitations, inspired by SGCN (Li et al., 2026) and ISGLNet (Zhang et al., 2025), the proposed LGFE-ResNet18 is designed as illustrated in **Figure 1**. LGFE modules are embedded into the four C2f stages of ResNet-18 to progressively compensate for feature attenuation at different network depths, thereby enabling effective extraction and integration of fine spatial details from shallow layers and high level semantic information from deeper layers. In the module design, the local texture branch calculates the standard deviation of pixel intensities within local regions. By exploiting the statistically distinguishable texture deviations between lesion regions and relatively homogeneous backgrounds, this branch adaptively strengthens feature responses at lesion boundaries and regions with abrupt intensity changes between diseased and healthy tissues. The global frequency domain branch maps the features into the frequency domain through a two dimensional discrete Fourier transform. Selective frequency band enhancement and phase preservation are then used to explicitly encode structural spectral characteristics associated with disease symptoms, thereby improving the discrimination of subtle frequency domain differences. The two branches jointly introduce spatially aware complementary priors at the feature extraction stage. This design allows the network to preserve spatial details while capturing discriminative representations of small disease targets, effectively reducing the information attenuation caused by successive downsampling. It also provides a more complete spatial and frequency domain representation for the subsequent cross scale fusion and attention enhancement modules.

**Figure 2:**
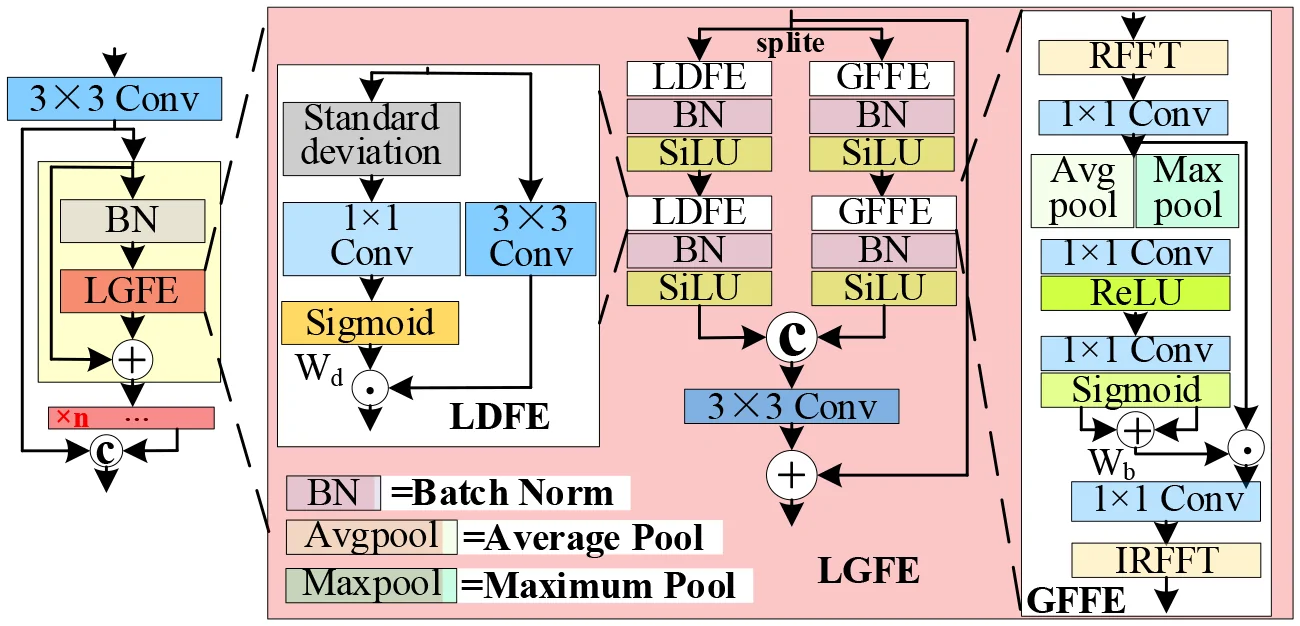
Network architecture of LGFE.

The detailed architecture of LGFE is illustrated in **Figure 2**. First, the input feature $X\in {\mathbb{R}}^{H\times W\times C}$ is partitioned along the channel dimension *C* and then processed by the local and global dual branch structure composed of LDFE and GFFE. LDFE extracts fine grained local texture variations associated with small lesions, whereas GFFE captures complementary global structural information. Through the joint modeling of these two branches, interference caused by complex backgrounds and occlusion is reduced, allowing the network to obtain more comprehensive and discriminative features for small disease targets. Finally, the features generated by the two branches are concatenated and passed through a 3×3 convolution to produce the LGFE enhanced output feature. Calculated as Equations 1, 2:

(1) $$F_{\text{LDFE}}=f_{\text{splite}}\left(X\right)\in {\mathbb{R}}^{H\times W\times C/2}$$

(2) $$F_{\text{GFFE}}=f_{\text{splite}}\left(X\right)\in {\mathbb{R}}^{H\times W\times C\left(1-\frac{1}{2}\right)}$$

where *F* denotes the output feature of each module, *F*_LDFE_ and *F*_GFFE_ represent the input features to LDFE and GFFE, respectively, *f* represents the corresponding operation, and *f*_splite_ denotes the channel wise feature splitting operation. The architecture of LDFE is illustrated in **Figure 2**. The module first extracts local deviation features from the input feature map. Specifically, a standard deviation operation is applied at each spatial position to calculate the pixel standard deviation within a 3×3 neighborhood. This process generates a deviation feature map that characterizes the intensity of local texture variations in citrus disease features. Calculated as Equation 3:

(3) $$F_{\text{Standard deviation}}\left(F_{\text{GFFE}}\right)=\sqrt{E\left[{F_{\text{GFFE}}}^{2}\right]-E{\left[F_{\text{GFFE}}\right]}^{2}+\epsilon }$$

The first moment, *E*[] represents the mean pixel value within the local window and is obtained by applying a 3×3 convolution to the input feature. The resulting deviation feature map is then fed into a dynamic gating structure. After passing through a 1×1 convolution and a Sigmoid activation function, the module adaptively assigns convolution kernels with different receptive fields and weights to individual spatial locations. Fine grained convolution is applied to regions with large local deviations, such as lesion boundaries, transition regions, and locations containing overlapping disease symptoms, to preserve edge and texture information. In contrast, smoother convolution is used in healthy regions or homogeneous background areas with small local deviations to reduce unnecessary computational cost. Through this adaptive design, LDFE strengthens the modeling of complex lesion morphologies across different citrus diseases and improves the localization accuracy and structural completeness of lesion boundaries.

The detailed architecture of GFFE is illustrated in **Figure 2**. First, the input feature $X\in {\mathbb{R}}^{H\times W\times C}$ is transformed into the frequency domain using the real fast Fourier transform, which makes the global frequency responses associated with disease patterns more explicit. The resulting complex frequency representation is separated into its real and imaginary components, and the spectrum is then divided into several subbands along the channel dimension. Different subbands encode distinct frequency information, among which some contain discriminative responses associated with disease symptoms. A frequency band enhancement network based on channel attention is subsequently introduced to identify and strengthen informative subbands. Global average pooling and global max pooling are applied to capture complementary global statistics from each subband. The pooled features are processed by two 1×1 convolutional layers with ReLU and Sigmoid activation functions. The outputs of the two pooling branches are then added to generate a weight coefficient within the range of [0,2] for each frequency band. A coefficient greater than 1 strengthens the corresponding subband, whereas a coefficient below 1 suppresses its response. After weight generation, the complete channel features of each subband are multiplied by their corresponding coefficients to achieve adaptive frequency enhancement or suppression. A 1×1 convolution is then applied to recouple all subbands into a complete frequency representation. Finally, the inverse real fast Fourier transform converts the refined frequency features back into the spatial domain. This design enables the network to distinguish disease textures from background noise according to their different responses across frequency bands. It dynamically emphasizes discriminative frequency components associated with lesion boundaries and leaf vein textures, thereby improving the resistance of the citrus disease detection model to interference from complex field environments during end to end training.

### 3.2 CAFF

**Figure 3:**
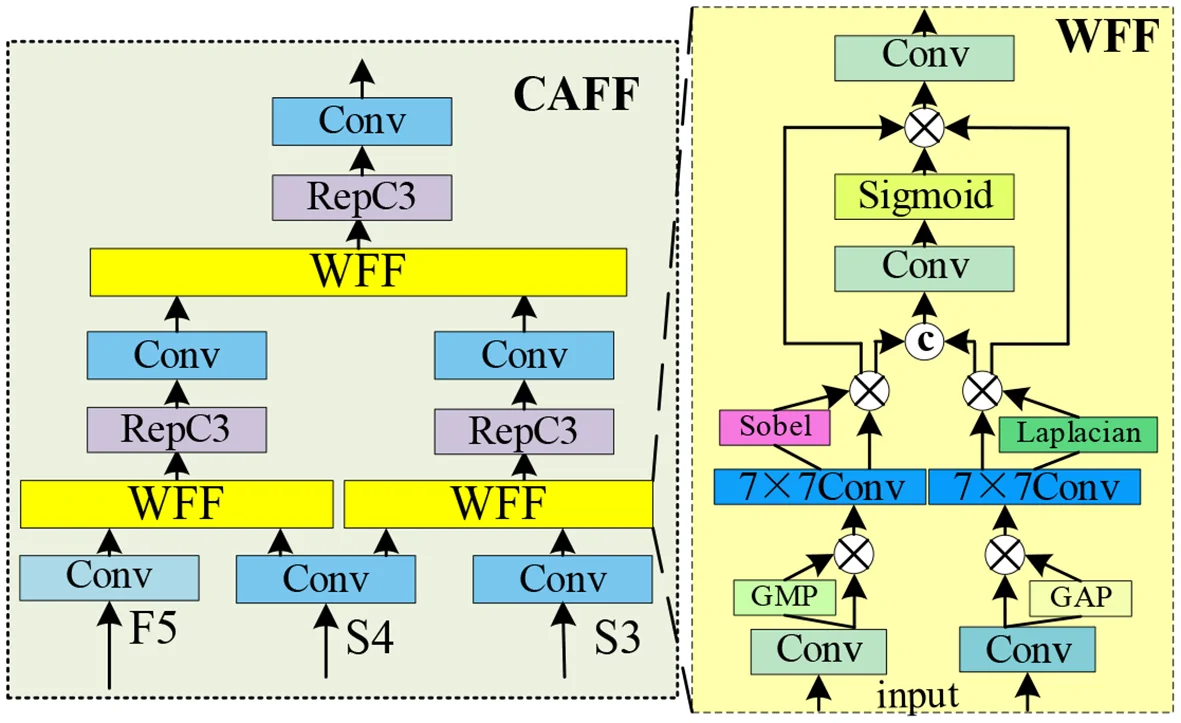
Network architecture of CAFF.

The baseline RT-DETR employs CCFM for cross scale feature fusion and interaction. When restoring the spatial resolution of multiscale features through upsampling, deterministic mappings based on nearest neighbor or bilinear interpolation cannot adaptively reconstruct high frequency details lost because of occlusion or insufficient illumination according to semantic information. From a signal processing perspective, stride 2 convolution and pooling during downsampling behave as low pass operations in the frequency domain, causing spatial high frequency components above the Nyquist frequency to be irreversibly removed. Although upsampling restores the spatial dimensions of the feature maps, it cannot recover the high frequency components that have already been discarded. Consequently, spatial structures such as small lesion boundaries and abrupt texture variations become blurred and are difficult to compensate for during subsequent feature fusion. In addition, the convolution based fusion in CCFM performs a fixed weight linear recombination of cross level features and lacks a dynamic discrimination mechanism based on feature confidence. As a result, background noise introduced by occluded regions is aggregated with genuine lesion texture responses using comparable weights, leading to strong coupling between noise and disease related features across both channel and spatial dimensions. During backpropagation, gradients induced by background noise may conflict with the genuine gradients associated with lesion boundaries, interfering with accurate boundary localization and regression. This ultimately leads to localization deviations and reduced confidence for occluded lesions. Inspired by DICFusion (Wang et al., 2026) and FusionMamba (Xie et al., 2024), a cross scale adaptive feature fusion module, termed CAFF, is designed, as illustrated in **Figure 3**. CAFF adopts a progressive layerwise fusion strategy and incorporates the advantages of RepC3 in gradient propagation and feature reuse to achieve adaptive aggregation of multilevel features from shallow to deep layers. For citrus disease detection under complex orchard conditions, CAFF progressively integrates shallow high resolution details with deep semantic information. This process compensates for the loss of spatial details caused by upsampling, strengthens the representation of small lesions and blurred boundaries, and improves sensitivity to mild symptoms at early disease stages. Moreover, the adaptive aggregation weights dynamically adjust the fusion coefficients according to the reliability of feature maps at different spatial locations and network levels. Lower weights are assigned to occluded regions and areas affected by background noise, thereby reducing the coupling between irrelevant interference and lesion features and improving feature discrimination under complex orchard conditions. Combined with the cross stage partial connections and enhanced gradient flow of RepC3, CAFF produces a multiscale feature pyramid with stronger discriminative capacity and improved spatial consistency while maintaining a relatively low computational cost. The refined feature representation provides a more reliable input for the subsequent decoder, reduces lesion localization deviations under occlusion, and establishes a robust feature basis for accurate citrus disease detection in complex orchard scenes.

CAFF takes the three feature maps F5, S4, and S3 produced by LGFE-ResNet18 as its inputs, as illustrated in **Figure 3**. First, the shallow features S4 and S3 are processed by 3×3 convolutions for downsampling and channel alignment, so that their spatial scales and channel dimensions match the requirements of subsequent cross scale feature fusion. In contrast, the deep feature F5 is directly used as the high level semantic branch for feature fusion. The aligned shallow features and the deep feature F5 are then fed into the WFF module for feature interaction and weighted fusion. During this process, WFF dynamically adjusts the fusion weights of features from different levels, thereby enhancing effective lesion responses while suppressing noise interference from occluded regions. After WFF based interaction, the features at each level are further strengthened and refined by RepC3. By exploiting cross stage partial connections and a multipath gradient flow design, RepC3 enriches semantic representation and preserves spatial details while maintaining computational efficiency. Finally, the refined features are progressively processed through successive RepC3 modules to achieve adaptive multilevel fusion from shallow to deep layers. In this way, CAFF generates multiscale feature maps that jointly preserve high resolution spatial details and strong semantic discrimination, thereby providing more reliable and informative features for the subsequent decoder.

The structure of WFF is illustrated in **Figure 3**. Taking S4 and F5 as an example, the MFM first applies 1×1 and 3×3 convolutions to the two input features, respectively, to align their channel dimensions. Global max pooling is then used to emphasize the most salient responses associated with disease regions, while global average pooling captures complementary global statistics related to lesion textures and fine details. The resulting features are subsequently integrated with the original inputs through skip connections, which preserves the initial feature information while enhancing disease related responses. Calculated as Equations 4–7:

(4) $$F_{\text{F}5}\prime =f_{1\times 1\text{Conv}}\left(F5\right)$$

(5) $$F_{\text{S}4}\prime =f_{3\times 3\text{Conv}}\left(S4\right)$$

(6) $$F_{\text{GMP}}=\sigma \left(W_{2}\cdot \delta \left(W_{1}\cdot f_{\text{GMP}}\left(F_{\text{F}5}\prime \right)\right)\right)$$

(7) $$F_{\text{GAP}}=\sigma \left(W_{2}\cdot \delta \left(W_{1}\cdot f_{\text{GAP}}\left(F_{\text{S}4}\prime \right)\right)\right)$$

where $f_{1\times 1\text{Conv}}$ and $f_{3\times 3\text{Conv}}$ denote the 1×1 and 3×3 convolution operations, respectively. σ and *δ* represent the Sigmoid and ReLU activation functions, respectively. *W*_2_ and *W*_1_ denote the weights of the fully connected layers, and · represents elementwise multiplication.

The refined features are then processed by a 7×7 convolution. Subsequently, the Laplacian operator, Sobel operator, and skip connection are jointly employed to extract complementary disease features. The Laplacian and Sobel operators strengthen edge responses and directional gradient information, respectively, while the skip connection preserves the original feature representation. This design effectively compensates for the loss of spatial details caused by upsampling and improves the representation of small lesions and regions with indistinct boundaries. Calculated as Equations 8–10:

(8) $$F_{\text{Laplance}}=\sigma \left(f_{7\times 7\text{Conv}}\left(f_{\text{Laplance}}\left(F_{\text{GAP}}\right)\right)\right)$$

(9) $$F_{\text{Sobel}}=\sigma \left(f_{7\times 7\text{Conv}}\left(f_{\text{Sobel}}\left(F_{\text{GMP}}\right)\right)\right)$$

(10) $$F_{\text{Cat}}=f_{\text{Concat}}\left[F_{\text{Laplance}}⊙F_{\text{GAP}};F_{\text{Sobel}}⊙F_{\text{GMP}}\right]$$

Subsequently, the fused feature is passed through a 1×1 convolution to generate the focused feature map. Calculated as Equation 11.

(11) $$F_{\text{Cat}}\prime =\sigma \left(f_{1\times 1\text{Conv}}\left(F_{\text{Cat}}\right)\right)$$

Finally, the two feature branches described above are integrated through adaptive weighted fusion. Calculated as Equation 12.

(12) $$F_{\text{CAFF}}=f_{1\times 1\text{Conv}}\left(F_{\text{Cat}}^{\prime }⊙F_{\text{Sobel}}+\left(1-F_{\text{Cat}}^{\prime }\right)⊙F_{\text{Laplance}}\right)$$

where ⊙ denotes the Hadamard product, and *f*_Concat_[]; represents the feature concatenation operation.

### 3.3 WDAM

The baseline RT-DETR employs self-attention to enhance the deep feature S5, thereby improving high level semantic representation through global contextual modeling and producing the enhanced feature F5. However, self-attention requires the construction of query *Q*, key *K*, and value *V* matrices, followed by computationally intensive matrix multiplications to establish global feature interactions. Its computational complexity increases quadratically with the spatial resolution of the feature map, resulting in substantial resource consumption and a heavier overall inference burden. Moreover, self-attention densely models interactions among all spatial positions and assigns comparable participation to disease regions and background areas during feature aggregation. In complex citrus orchard scenes, lesion regions often exhibit relatively weak semantic responses in deep feature maps, whereas large leaf areas, prominent vein textures, and uneven illumination tend to dominate global attention computation. Consequently, a considerable proportion of the available computational resources is consumed by irrelevant background information, while discriminative lesion features become entangled with redundant contextual responses during global encoding. This form of global feature enhancement fails to sufficiently emphasize subtle lesion patterns and may further weaken disease related representations because of excessive background involvement. As a result, detection performance can deteriorate under challenging conditions involving occlusion, illumination variation, and diverse lesion morphologies.

**Figure 4:**
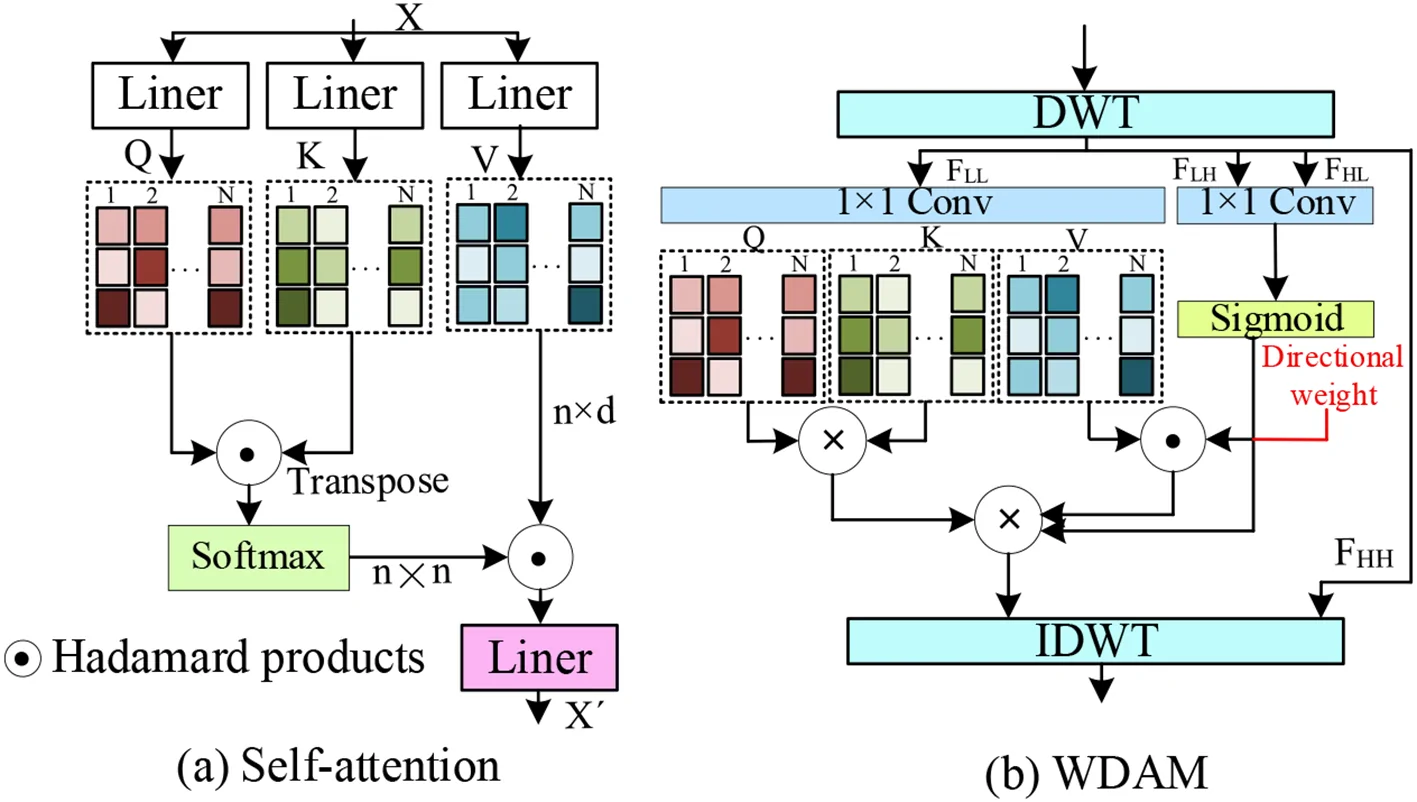
Network architectures of self-attention and WDAM. **(a)** Self-attention; **(b)** WDAM.

Inspired by Transformer (Vaswani et al., 2017) and Flickerformer (Qu et al., 2026), the WDAM module is designed as illustrated in **Figure 4**. In terms of feature discrimination, its directional modulation mechanism guides the network to focus on lesion boundaries and regions with abrupt texture variations, rather than assigning comparable attention to all spatial positions through dense global encoding. This design alleviates the dilution of disease related responses caused by conventional self-attention and strengthens the representation of small lesions and lesions with irregular morphologies. In terms of resistance to interference, wavelet decomposition separates horizontal and vertical directional responses from diagonal high frequency components. This separation enables the attention mechanism to better distinguish disease related textures from interfering patterns caused by leaf veins and uneven illumination. It also reduces the coupling between background noise and lesion features in occluded regions, thereby improving the robustness of feature discrimination under complex orchard conditions.

As shown in **Figure 4a**, self-attention projects the input feature *X* into the *K*, *Q*, and *V* matrices. The weight matrices along the *K* and *Q* directions are then multiplied to compute the attention weights, which are subsequently fused with the matrix along the *V* direction to produce the self-attention enhanced output. Calculated as Equation 13.

(13) $$F_{\text{Self-attention}}=\beta \left(QK^{T}/\sqrt{d}\right)⊙V$$

where *d* denotes the number of tokens, and *β* represents the Softmax activation function.

As shown in **Figure 4b**, WDAM applies the Haar discrete wavelet transform to the input feature $X\in {\mathbb{R}}^{H\times W\times C}$, decomposing it into one low frequency component $F_{LL}\in {\mathbb{R}}^{\frac{H}{2l+1}\times \frac{W}{2l+1}\times 2^{l}C}$ and three high frequency components *F_LH_*, *F_HL_*, and *F_LL_*. Calculated as Equation 14.

(14) $$\left[F_{\text{LL}},F_{\text{HL}},F_{\text{LH}},F_{\text{HH}}\right]=f_{\text{DWT}}\left(X\right)$$

where *F*_LH_, *F*_HL_, and *F*_HH_ denote the horizontal, vertical, and diagonal high frequency components, respectively, while *F*_LL_ represents the low frequency component. *f*_DWT_ denotes the discrete wavelet transform operation. Subsequently, the corresponding *K*, *Q*, and *V* matrices $Q=F_{\text{LL}}W^{Q}$, $K=F_{\text{LL}}W^{K}$, $V=F_{\text{LL}}W^{V}$ are computed from the decomposed features. These matrices are then processed by a 1×1 convolution followed by a Sigmoid activation function to generate the directional weights *F*_HL_ and *F*_LH_. Calculated as Equation 15.

(15) $$W=\sigma \left(f_{1\times 1\text{Conv}}\left[F_{\text{HL}},F_{\text{LH}}\right]\right)$$

Finally, the output feature of WDAM is reconstructed through the inverse discrete wavelet transform as follows. Calculated as Equation 16.

(16) $$F_{\text{WDAM}}=\xi \left(\frac{QK^{T}}{\sqrt{d}}+B\right)\left(W⊙V\right)$$

where *ξ* denotes the Softmax activation function.

### 3.4 Loss function

The baseline RT-DETR performs object detection through bounding box regression. Citrus lesions are often small, irregularly shaped, and characterized by indistinct boundaries. In such cases, IoU and its variants, including GIoU, CIoU, and DIoU, are highly sensitive to positional deviations of small targets. When the overlap between the predicted box and the ground truth lesion box is limited, the resulting regression gradients may become extremely weak. In addition, IoU based loss functions do not adequately account for differences in sample difficulty and lack a targeted gradient adjustment mechanism for heavily occluded lesions or disease regions with diverse morphologies, thereby limiting bounding box regression accuracy. Focaler Shape IoU improves regression by using the linear interval mapping of Focaler IoU to adaptively emphasize samples of different difficulty levels and by incorporating bounding box geometry through Shape IoU. However, it remains fundamentally dependent on the IoU framework. Therefore, it does not fully address the optimization difficulty associated with small lesions, for which minor positional deviations can cause substantial IoU fluctuations because of their extremely limited pixel area. To address these limitations, Gaussian Combined Distance, termed GCD (Guan et al., 2025), is introduced into the loss function of LCWG-DETR as the bounding box similarity measure and regression objective. GCD models each bounding box as a two dimensional Gaussian distribution and characterizes its spatial properties using the center distance and covariance matrix. This formulation provides stronger regression gradients for lesions with extremely small spatial dimensions or elongated shapes, thereby alleviating gradient attenuation and scale sensitivity during small target regression. Furthermore, GCD applies a nonlinear mapping to normalize the distance measure to the interval [0,1], which maintains smooth and controllable gradients during optimization. The regression strength can therefore be adjusted according to the localization accuracy of each predicted lesion, improving the localization of subtle early stage citrus lesions and the overall detection performance. The squared distance used in GCD is expanded as follows. Calculated as Equations 17, 18.

(17) $${\text{D}}_{gc}^{2}\left(N_{p},N_{t}\right)=\frac{1}{2}\left(\frac{{\left(x_{p}-x_{t}\right)}^{2}}{m_{p}^{2}}+\frac{{\left(y_{p}-y_{t}\right)}^{2}}{n_{p}^{2}}\right)+\frac{1}{2}\left(\frac{{\left(m_{p}-m_{t}\right)}^{2}}{4m_{p}^{2}}+\frac{{\left(n_{p}-n_{t}\right)}^{2}}{4n_{p}^{2}}\right)+\frac{1}{2}\left(\frac{{\left(x_{t}-x_{p}\right)}^{2}}{m_{t}^{2}}+\frac{{\left(y_{t}-y_{p}\right)}^{2}}{n_{t}^{2}}\right)+\frac{1}{2}\left(\frac{{\left(m_{t}-m_{p}\right)}^{2}}{4m_{t}^{2}}+\frac{{\left(n_{t}-n_{p}\right)}^{2}}{4n_{t}^{2}}\right)$$

(18) $$L_{\text{GCD}}=exp\left(-\sqrt{{\text{D}}_{gc}^{2}\left(N_{p},N_{t}\right)}\right)$$

where ${\text{D}}_{gc}^{2}\left(N_{p},N_{t}\right)$ denotes the squared GCD distance between the Gaussian distribution *N_p_* of the predicted bounding box and the Gaussian distribution *N_t_* of the ground truth bounding box. *N_p_* and *N_t_* represent the two dimensional Gaussian distributions corresponding to the predicted and ground truth bounding boxes, respectively. *x_p_* and *y_p_* denote the horizontal and vertical coordinates of the center of the predicted bounding box, while *m_p_* and *n_p_* represent its width and height, respectively. Similarly, *x_t_* and *y_t_* denote the horizontal and vertical coordinates of the center of the ground truth bounding box, and *m_t_* and *n_t_* represent its width and height, respectively. All coordinate and size values are measured in pixels. *L*_GCD_ denotes the final similarity measure used for label assignment or loss calculation, with a value range of [0,1]. A larger value indicates greater similarity between the two bounding boxes, and *L*_GCD_ = 1 indicates complete overlap. exp denotes the natural exponential function, while exp performs the nonlinear mapping of the distance to the interval [0,1]. ${\text{D}}_{gc}^{2}$ denotes the square root operation applied to the squared distance, yielding a distance value with the same dimensional unit as the bounding box coordinates.

## 4 Experimental results and analysis

### 4.1 Dataset description

**Figure 5:**
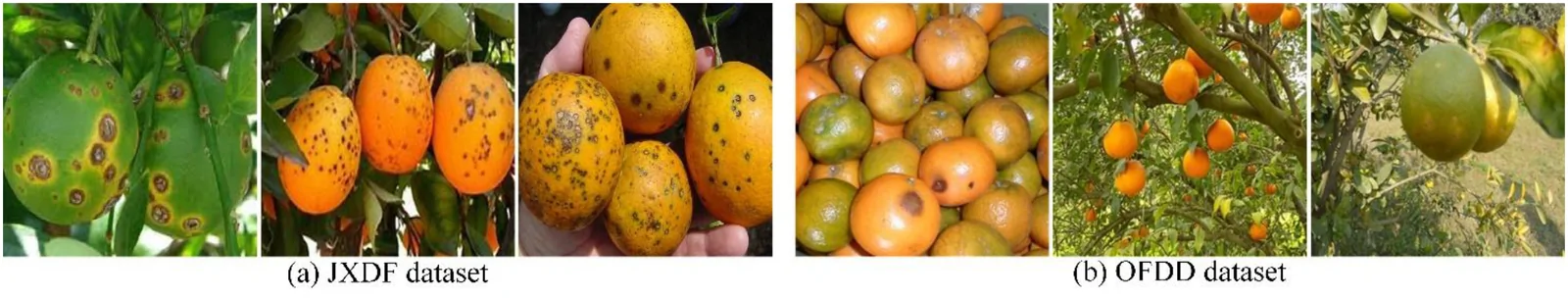
Representative samples from the datasets. **(a)** JXDF dataset; **(b)** OFDD dataset.

To evaluate the effectiveness and generalization ability of the proposed LCWG-DETR, experiments were conducted on the self-collected Jiangxi Citrus Fruit Diseases Dataset, termed JXDF (Gao et al., 2026), and the public Orange Fruit Diseases Dataset, termed OFDD (Faisal et al., 2023). Representative samples from the two datasets are shown in **Figure 5**. OFDD is a publicly available citrus fruit disease image dataset compiled by Faisal et al. in 2023 from two public sources, Mendeley Data and Kaggle, followed by data cleaning and reorganization. The dataset contains 700 citrus fruit images covering five categories: Black Spot (Phyllosticta citricarpa), Canker (Xanthomonas citri subsp. citri), Greening (Candidatus Liberibacter asiaticus), Healthy, and Scab (Elsinoë fawcettii). Disease regions in each image were annotated with bounding boxes using CVAT, and all annotations were converted into the YOLO format for direct use in object detection tasks. The images were collected at multiple farm locations under orchard conditions using mobile phone cameras at a resolution of 800×800 pixels. They include diverse natural illumination conditions, such as front lighting, backlighting, and diffuse illumination, as well as complex backgrounds involving soil, branches, leaves, and sky. Variations in fruit orientation are also represented, allowing the dataset to preserve detailed disease characteristics while reflecting the visual diversity of real orchard environments. The main challenges of OFDD arise from the high similarity in color and morphology among the early symptoms of different diseases, particularly between black spot and scab. Substantial illumination variation may also reduce the stability of color features, while branches, leaves, soil, and other background elements introduce considerable interference. In addition, the same disease may exhibit markedly different visual characteristics at different developmental stages. These factors place high demands on the robustness and resistance to interference of detection models. Therefore, OFDD is suitable for citrus fruit disease classification and detection, object detection model development, performance comparison, and transfer learning. It also provides a consistent evaluation benchmark for disease recognition and visual perception systems in citrus harvesting robots.

The JXDF (Jiangxi Citrus Disease Dataset) was constructed specifically to address visual perception challenges faced by citrus harvesting robots in complex orchard environments. The dataset was collected primarily from real production environments in Ganzhou, Jiangxi Province, China, one of the country’s major production regions for navel oranges and citrus fruits, and was supplemented with a small number of citrus images collected from online sources. Ganzhou is located in a humid subtropical monsoon climate zone. In 2021, the citrus planting area in Jiangxi Province reached 20 million mu, ranking first nationwide, while navel orange production reached 17 million tons, ranking second in China. Selecting this region as the main data collection site therefore provides strong industrial representativeness, disease diversity, and environmental complexity for the dataset. Data acquisition was mainly conducted from May to September each year. This period corresponds to the hot and humid season in southern Jiangxi, during which various citrus diseases enter their high incidence and symptom expression stages and lesion characteristics become particularly evident. At the same time, citrus fruits develop from the expansion stage toward the early ripening stage, during which peel color and texture exhibit substantial variation. These conditions provide diverse samples for training disease detection models. The dataset contains five citrus fruit categories: Orange-Greening, Orange-Melanose (Diaporthe citri);, Orange-Black-Spot, Orange-Canker, and Orange-Healthy. These categories are commonly observed in the citrus producing areas of southern Jiangxi. In addition, the early symptoms of some diseases exhibit high visual similarity to healthy citrus peel, posing considerable challenges for conventional visual detection methods.

To enhance dataset diversity and model generalization, several key variables were systematically varied during data acquisition. Imaging distances covered three scale ranges: close range (0.3–0.8m), medium range (0.8–2.0m), and long range (2.0–5.0m). Citrus fruits were captured from multiple viewpoints, including frontal, lateral, and rear views. The illumination conditions included front lighting, backlighting, and natural diffuse lighting commonly encountered in orchards. Backgrounds covered both natural orchard elements, such as soil, weeds, fallen leaves, branches, and foliage, and different fruit distributions ranging from isolated single fruits to densely clustered fruits. In addition, data were collected in two representative environments: orchards and packaging and distribution centers. Orchard scenes are characterized by complex backgrounds and highly variable illumination, whereas packaging and distribution environments generally provide cleaner and more controlled conditions. Together, these acquisition settings simulate the complete operational process of citrus harvesting robots from field harvesting to postharvest sorting, providing a diverse data basis for improving model robustness throughout the perception, decision, and execution workflow.

During dataset construction, the raw images were first subjected to a systematic quality screening process. Images blurred by camera shake, images with substantial feature loss caused by severe backlighting, and invalid samples in which targets were excessively occluded and could not be reliably annotated were removed. After this three stage quality screening process, 1,350 high quality citrus fruit images were retained. The citrus targets were then carefully annotated using LabelMe, and the annotations were converted into TXT files compatible with the YOLO format. To ensure annotation consistency and reliability, four annotation criteria were established. First, each bounding box was required to tightly enclose the target fruit to ensure accurate localization. Second, category labels were determined by jointly considering peel color uniformity, lesion morphology, including punctate, patchy, or depressed regions, and the clarity of lesion boundaries. Third, for partially occluded targets, only the visible regions were annotated to avoid annotation noise caused by subjective inference. Finally, for difficult samples exhibiting mixed symptoms of multiple diseases or ambiguous visual characteristics, one expert in plant pathology and one expert in computer vision jointly reviewed the samples and reached a final consensus, thereby improving the objectivity and consistency of category assignment.

After annotation and quality screening, both JXDF and OFDD were divided into training, validation, and test sets at a ratio of 8:1:1. JXDF contains 1,080 training images, 135 validation images, and 135 test images, whereas OFDD contains 560 training images, 70 validation images, and 70 test images. JXDF and OFDD differ substantially in image sources, acquisition devices, illumination conditions, and background complexity. JXDF was collected from real harvesting environments in citrus orchards in southern Jiangxi and covers multiple imaging distances, including close, medium, and long range views, as well as different fruit orientations. Its complex backgrounds and frequent target occlusions provide a suitable basis for evaluating model robustness and resistance to environmental interference in unstructured orchard scenes. In contrast, OFDD integrates images from multiple public data sources and is therefore suitable for assessing cross domain generalization. Joint evaluation on these two datasets provides a systematic benchmark for comprehensively assessing citrus disease detection methods in terms of scene diversity, disease category coverage, and robustness to environmental interference.

### 4.2 Experimental setup and evaluation metrics

To comprehensively evaluate the effectiveness of LCWG-DETR for citrus disease detection, all models were trained and evaluated under identical experimental conditions. The experiments were implemented using PyTorch 2.3.1 and Python 3.8.19 on a hardware platform equipped with an Intel Core i5-14600KF processor and an NVIDIA GeForce RTX 4080 Super GPU. The input image resolution was uniformly set to 640×640 pixels. Adam was used as the optimizer, with an initial learning rate of 0.001, a weight decay of 0.0005, and a momentum of 0.937. This configuration was selected because Adam combines adaptive gradient adjustment with momentum based optimization, providing stable convergence while effectively handling sparse gradients commonly encountered in detection tasks. The batch size and number of workers were both set to 4, and all models were trained for 200 epochs. This setting was determined through preliminary experiments by systematically examining the loss curves and validation mAP convergence trends of different models. The results showed that 200 epochs provided sufficient training iterations for all methods to converge close to their optimal performance. Using the same training duration also ensured a consistent and fair comparison among architectures with different convergence characteristics. In addition, no pretrained weights were used for any model, thereby eliminating the influence of prior knowledge from external datasets and ensuring that performance differences primarily originated from the network architectures and optimization strategies themselves. These unified experimental settings provide a fair and reliable basis for comparison among all baseline methods.

To comprehensively evaluate model performance, the experiments assess three aspects: detection accuracy, model complexity, and real time performance. Detection accuracy is measured using the average precision for each category AP, the mean average precision across all categories mAP, the mean average precision over multiple IoU thresholds mmAP or mAP@[0.5:0.95], and recall R. Higher values of these metrics indicate greater detection accuracy and stability, with fewer false detections and missed targets. Model complexity is evaluated using the number of parameters Para, and the number of floating point operations GFLOPs. A smaller parameter count and lower computational cost are more favorable for lightweight deployment. Real time performance is measured using frames per second, termed FPS. A model is considered capable of real time detection when its processing speed reaches at least 30 FPS, and a higher FPS indicates stronger real time processing capability.

### 4.3 Effectiveness analysis of the loss function

**Figure 6:**
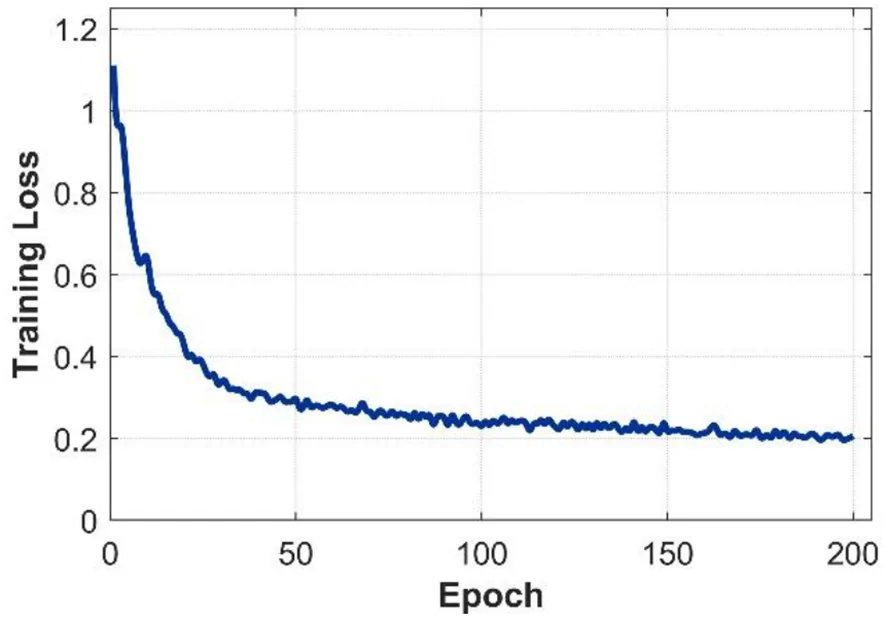
Training total loss curves.

To verify the effectiveness of the proposed loss function, the total training loss curve is presented in **Figure 6**. The total loss is composed of the classification loss, regression loss, and GCD loss, and therefore reflects the overall convergence behavior of the model during training. The relatively smooth downward trend indicates stable optimization of both classification and localization objectives and suggests that the different loss components remain well coordinated throughout the training process.

As shown in **Figure 6**, LCWG-DETR exhibits a smooth and stable decrease in training loss over 200 epochs. The loss decreases from an initial value of approximately 1.15 to below 0.2, without obvious oscillation, divergence, or prolonged stagnation, indicating stable parameter optimization and convergence. During the early training stage, the loss decreases rapidly as the model parameters move from random initialization toward a more effective solution. This behavior is consistent with the gradient characteristics introduced by GCD for small target regression. For early stage lesions with extremely small spatial dimensions, GCD models the bounding boxes as two dimensional Gaussian distributions and measures their discrepancy through both center distance and distributional differences. Consequently, even small localization deviations can produce effective regression gradients, alleviating the weak or vanishing gradients that may occur with IoU based losses when small predicted and ground truth boxes have little or no overlap. During the middle and late stages of training, the loss decreases more gradually. Over the final 100 epochs, it declines from approximately 0.24 to 0.14 while maintaining a smooth convergence trend. The scale aware distribution modeling of GCD helps maintain effective regression supervision for targets with different spatial sizes, reducing excessive optimization bias toward larger lesions. This property is particularly suitable for the citrus disease dataset, in which lesion sizes vary considerably. Overall, the convergence behavior in **Figure 6** is consistent with the intended design of GCD for dense small targets, large scale variations, and high localization requirements. These results support the effectiveness of GCD as the bounding box regression loss in LCWG-DETR and provide a stable optimization basis for accurate citrus disease localization.

### 4.4 Ablation study

To evaluate the effectiveness of each proposed component for citrus disease detection, ablation experiments were conducted on the JXDF and OFDD datasets, as reported in **Table 1**.

**Table 1:** Comparison of ablation experiment results.

Datasets	Method	LGFE	CAFF	WDAM	GCD	mAP/%	mmAP/%	R/%	Para/M	GFLOPs	FPS frame/s
JXDF	RT-DETR					93.17	88.74	91.48	20.09	58.34	85.71
		✓				95.86	91.29	93.27	**15.92**	**51.04**	**107.68**
		✓	✓			97.55	92.92	94.85	20.12	52.08	90.37
		✓	✓	✓		98.07	93.54	95.47	19.74	55.05	92.45
		✓	✓	✓	✓	**98.24**	**94.18**	**95.96**	19.74	55.05	92.45
OFDD	RT-DETR					92.87	77.26	90.58	20.09	58.34	85.71
		✓				94.39	79.55	92.37	**15.92**	**51.04**	**107.68**
		✓	✓			95.14	81.96	93.03	20.12	52.08	90.37
		✓	✓	✓		95.96	82.37	94.15	19.74	55.05	92.45
		✓	✓	✓	✓	**96.28**	**82.74**	**94.58**	19.74	55.05	92.45

Bold indicates the optimal result.

The ablation results in **Table 1** show that LCWG-DETR achieves the highest mAP, mmAP, and recall on both datasets. On JXDF, compared with the baseline model, LCWG-DETR improves mAP, mmAP, recall, and FPS by 5.07%, 5.44%, 4.48%, and 6.74 PFS, respectively, while decreasing Para and GFLOPs by only 0.35 MB and 3.29. Each proposed component therefore contributes positively to the overall detection performance. On OFDD dataset, LCWG-DETR improves mAP, mmAP, recall, and FPS by 3.41%, 5.48%, 4.00%, and 6.74 frames per second, respectively, with the same decreases of 0.35 MB in Para and 3.29 in GFLOPs. The performance gains can be attributed to the complementary roles of the four proposed components. LGFE-ResNet18 enhances local deviation textures and global structural information in the Fourier frequency domain, thereby compensating for the progressive weakening of small lesion features caused by stacked convolution and pooling operations. This design improves the localization of disease regions while suppressing interference from complex backgrounds and occlusion. CAFF progressively integrates shallow features with high spatial resolution and deep features with strong semantic information through cross scale adaptive weighting, reducing the loss of spatial details and the coupling of lesion features with background noise during upsampling. WDAM replaces self attention for enhancing the deep feature S5. Through Haar wavelet decomposition and directional modulation, it guides the network to focus on lesion boundaries and regions with abrupt texture variations, strengthening small lesion representations while maintaining real time inference capability. GCD models bounding boxes as two dimensional Gaussian distributions and performs regression using the center distance and covariance information. It provides stronger gradient responses for lesions with extremely small spatial dimensions, thereby alleviating gradient attenuation and scale sensitivity in small target regression. These four components work together to improve the detection accuracy and robustness of LCWG-DETR for small and occluded lesions. The results confirm the effectiveness and practical value of the proposed design for citrus disease detection in complex orchard environments.

**Figure 7:**
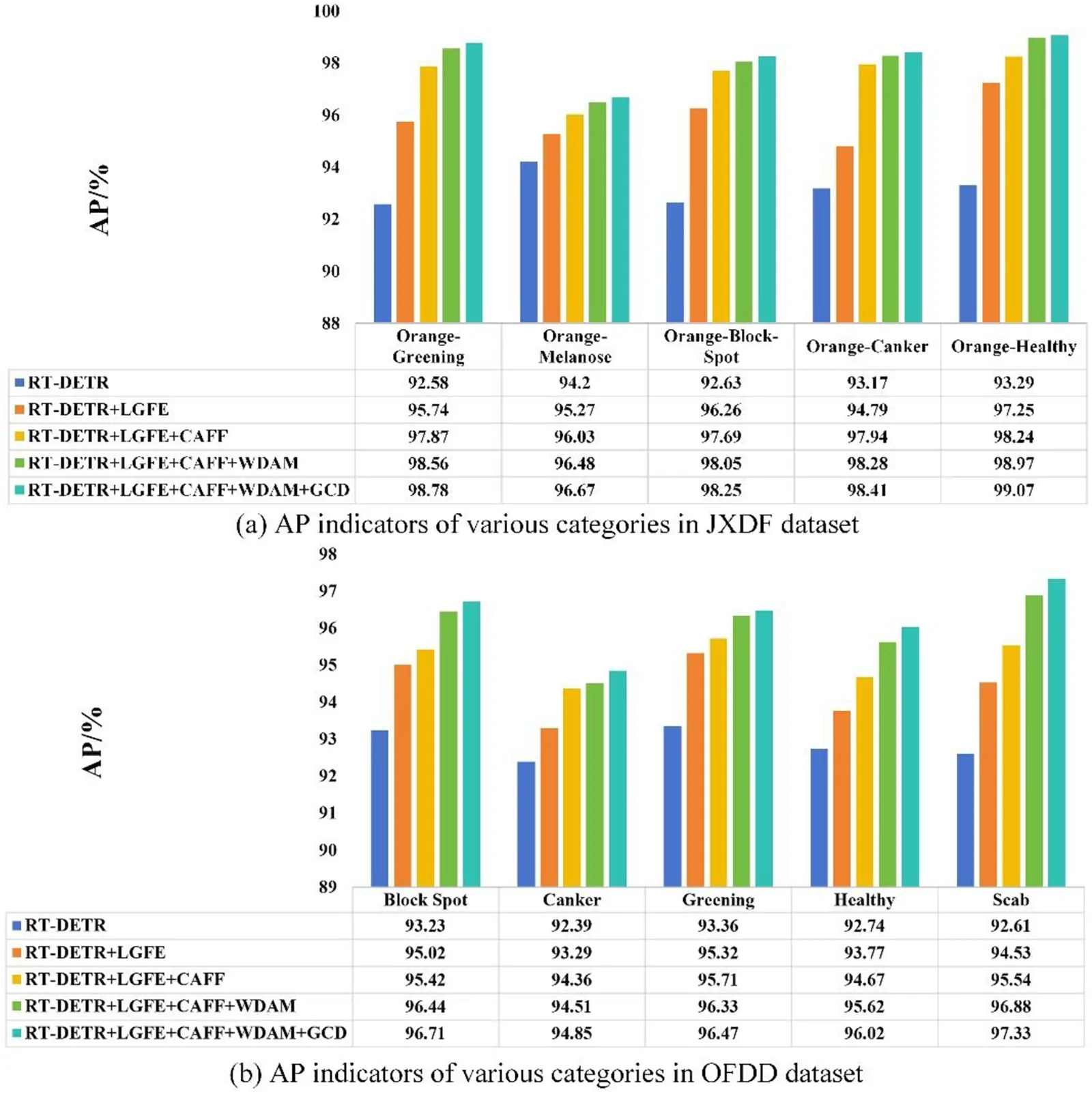
Ablation results for the AP of each category. **(a)** AP indicators of various categories in JXDF dataset; **(b)** AP indicators of various categories in OFDD dataset.

To examine the effect of each proposed component on individual disease categories, the AP results are presented in **Figure 7**. LCWG-DETR consistently outperforms the baseline RT-DETR on both JXDF and OFDD. On JXDF, the baseline model achieves its lowest AP for the Greening category. In real orchard scenes, greening symptoms often exhibit weak boundary contrast, and their subtle texture patterns can become highly entangled with those of healthy fruit in deep feature maps. Consequently, self attention based global contextual modeling has difficulty forming sufficiently discriminative spatial responses. Introducing LGFE-ResNet18 increases the AP of Greening and Healthy by 3.16% and 3.96%, respectively. By combining local deviation guided texture modeling with frequency domain structural encoding, LGFE-ResNet18 strengthens subtle lesion features at the early feature extraction stage and compensates for the attenuation of high frequency information caused by stacked convolution operations. Adding CAFF further improves the AP of Black-Spot and Canker by 1.43% and 3.15%, respectively. Through progressive multilevel fusion and adaptive weighting, CAFF strengthens disease related responses in occluded regions and introduces detailed shallow features into the feature pyramid. This process preserves lesion boundaries while reducing the coupling between lesion features and background noise during cross scale fusion, thereby improving feature discrimination under complex orchard conditions. WDAM provides additional improvements for Greening and Healthy. The module derives directional modulation weights from the horizontal and vertical high frequency components obtained through Haar wavelet decomposition. These weights are applied to the value features through elementwise multiplication, encouraging the network to prioritize lesion boundaries with clear directional texture variations. This mechanism suppresses interference from complex backgrounds and reduces the dilution of weak disease responses during feature aggregation. After GCD is introduced, the AP values for Orange-Greening, Orange-Melanose, Orange-Black-Spot, Orange-Canker, and Orange-Healthy reach 98.78%, 96.67%, 98.25%, 98.41%, and 99.07%, respectively. By assigning stronger regression gradients to center deviations in extremely small lesions, GCD guides the predicted bounding boxes toward more accurate locations and further improves category level detection performance. LCWG-DETR also demonstrates strong performance on OFDD. LGFE-ResNet18 improves the AP of Black Spot and Greening by 1.79% and 1.96%, respectively, indicating that its sensitivity to subtle texture abnormalities generalizes across different data distributions. CAFF and WDAM provide the most evident improvement for Scab. The rough surfaces and wart like protrusions of scab lesions produce pronounced multidirectional texture variations, which generate strong responses in the horizontal and vertical high frequency wavelet subbands and are therefore well matched to the directional enhancement mechanism of WDAM. Overall, the category level ablation results on both datasets demonstrate that the proposed components provide complementary improvements and retain their effectiveness across different disease categories and data distributions.

**Figure 8:**
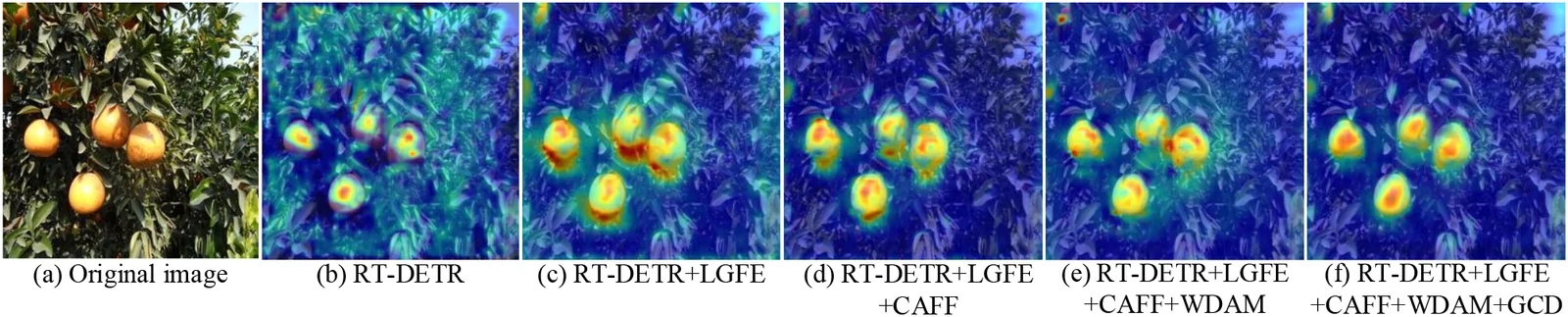
Feature heatmaps for the ablation experiments. **(a)** Original image; **(b)** RT-DETR; **(c)** RT-DETR+LGFE; **(d)** RT_DETR+LGFE+CAFF; **(e)** RT-DETR+LGFE+CAFF+WDAM; **(f)** RT-DETR+LGFE+CAFF+WDAM+GCD0.

To visually demonstrate the degree to which each proposed component focuses on disease regions in complex citrus disease detection, one image was randomly selected from the JXDF dataset and used for feature heatmap visualization. The spatial response distributions are shown in **Figure 8**.

The baseline RT-DETR produces spatially dispersed heatmap responses. In addition to disease regions, the model also activates healthy peel surfaces, branches, leaves, and specular highlights. Consequently, disease related information is diluted during global encoding, making it difficult to form precise attention on lesion regions. After introducing LGFE-ResNet18, the responses become more concentrated around disease regions, with stronger activation over small lesions and subtle texture variations. This improvement results from the combination of local deviation guided texture modeling and frequency domain structural encoding, which strengthens weak lesion features. However, some responses remain on healthy peel wrinkles and regions with abrupt illumination changes, indicating that improved feature extraction alone cannot completely eliminate interference from complex backgrounds. With the addition of CAFF, the heatmap becomes more focused on lesion contours and boundaries, while responses from adjacent healthy peel and occluded background regions are reduced. This result shows that progressive cross scale aggregation and adaptive weighting effectively separate disease features from background noise and establish clearer spatial discrimination. After WDAM is introduced, high responses are concentrated more accurately on lesion boundaries and regions with abrupt texture changes, whereas specular highlights and foliage occlusion receive relatively weak activation. This observation confirms that the directional modulation guided by Haar wavelet decomposition improves sensitivity to horizontal and vertical texture variations while suppressing nondirectional background responses. Finally, after GCD is incorporated into the regression objective, LCWG-DETR presents the most concentrated and discriminative response distribution. The highly activated regions correspond closely to the actual lesion locations, and their spatial coverage shows better agreement with the lesion contours than those of the other ablation variants. Although GCD does not directly generate attention features, its more stable localization supervision further guides the network toward disease relevant regions during training. These results demonstrate that LGFE-ResNet18, CAFF, WDAM, and GCD provide complementary improvements in feature extraction, cross scale fusion, deep feature enhancement, and bounding box regression. Together, they establish a complete perception and discrimination process that suppresses background interference and progressively refines lesion localization from coarse regional responses to precise fine grained focus in complex orchard environments.

### 4.5 Comparative experiments

**Figure 9:**
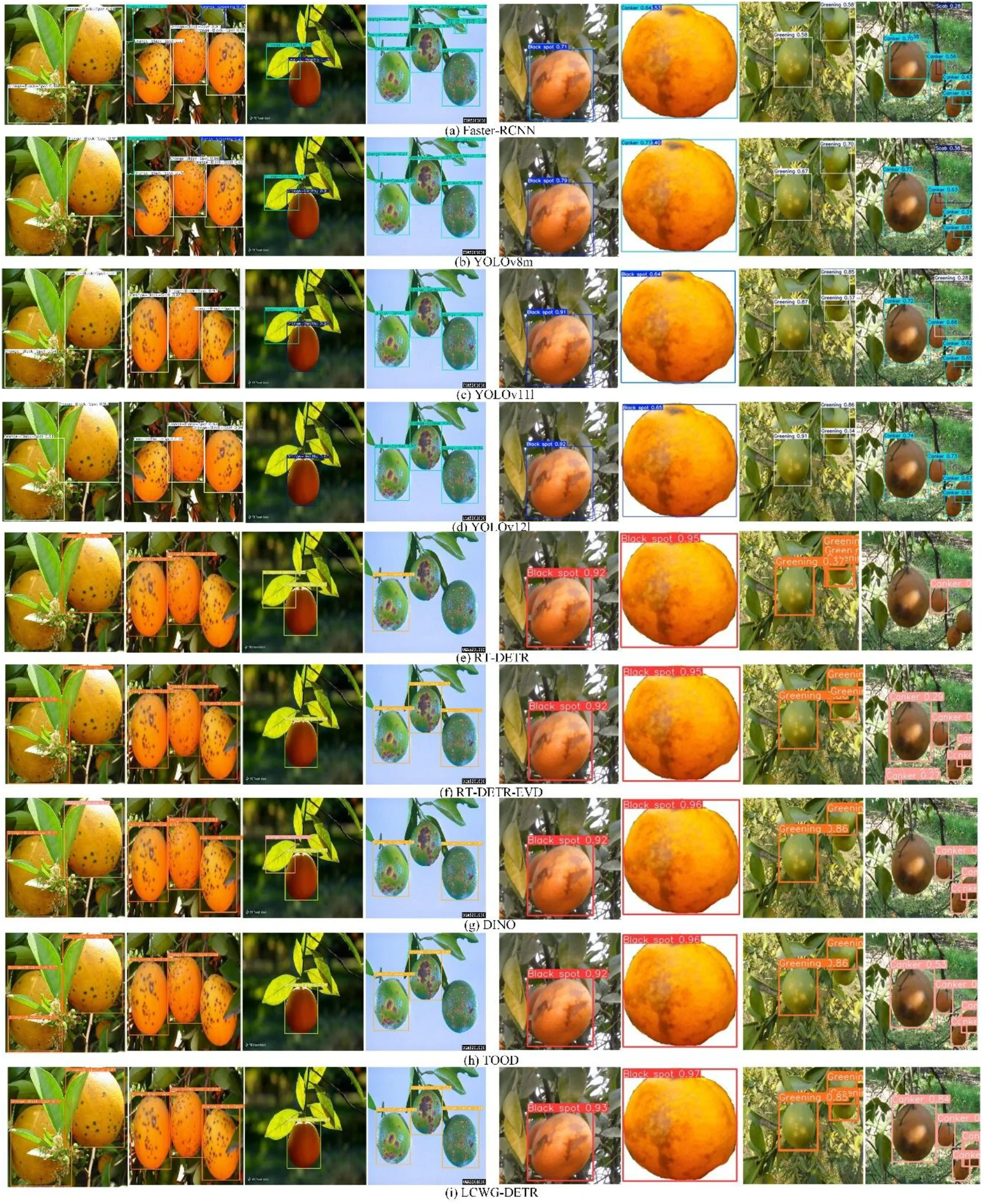
Visual comparison of detection results obtained by different methods. **(a)** Faster-RCNN; **(b)** YOLOv8m; **(c)** YOLOv11l; **(d)** YOLOv12l; **(e)** RT-DETR; **(f)** RT-DETR-ECD; **(g)** DINO; **(h)** TOOD; **(i)** LCWG-DETR.

To evaluate the effectiveness of LCWG-DETR for citrus disease detection under complex environmental conditions, eight representative methods were selected for comparison. These include Faster R-CNN (Chen and Gupta, 2017), YOLOv8m (Varghese and M., 2024), YOLOv11l (Khanam and Hussain, 2024), YOLOv12l (Tian et al., 2025), RT-DETR (Zhao et al., 2024), TOOD (Feng et al., 2021), DINO (Ren et al., 2025), and the dedicated citrus disease detection method RT-DETR-EVD (Hu et al., 2025). The selected models cover three mainstream detection paradigms at the architectural level, namely two stage CNN based detectors, one stage CNN based detectors, and end to end Transformer based detectors. This selection provides a comprehensive comparison of different technical approaches for citrus disease detection. In terms of practical applicability, all selected methods have been widely adopted in agricultural visual detection and are supported by publicly available implementations, thereby ensuring the fairness and reproducibility of the comparative experiments. The comparative results are presented in **Table 2** and **Figure 9**.

**Table 2:** Comparison of experimental results obtained by different methods.

Datasets	Methods	mAP/%	mmAP/%	R/%	Para/M	GFLOPs	FPS frame/s
JXDF	Faster-RCNN	91.23	90.04	88.64	54.12	93.33	28.42
	YOLOv8m	94.15	91.57	89.61	25.91	78.91	88.48
	YOLOv11l	94.37	91.89	90.37	25.37	86.88	79.46
	YOLOv12l	95.88	92.41	93.19	26.44	88.92	75.59
	RT-DETR	93.17	88.74	91.48	20.09	58.34	85.71
	RT-DETR-EVD	96.14	92.81	94.38	**14.23**	**49.51**	**108.17**
	DINO	96.32	92.59	94.86	22.24	91.70	73.24
	TOOD	94.24	92.16	93.49	32.03	123.04	54.38
	LCWG-DETR	**98.24**	**94.18**	**95.96**	19.74	55.05	92.45
OFDD	Faster-RCNN	89.34	73.39	88.65	54.12	93.33	28.42
	YOLOv8m	92.19	75.89	89.74	25.91	78.91	88.48
	YOLOv11l	92.76	77.18	90.42	25.37	86.88	79.46
	YOLOv12l	93.21	77.43	90.84	26.44	88.92	75.59
	RT-DETR	92.87	77.26	90.58	20.09	58.34	85.71
	RT-DETR-EVD	94.85	80.39	92.04	**14.23**	**49.51**	**108.17**
	DINO	93.88	78.15	91.27	22.24	91.70	73.24
	TOOD	92.14	76.48	90.07	32.03	123.04	54.38
	LCWG-DETR	**96.28**	**82.74**	**94.58**	19.74	55.05	92.45

Bold indicates the optimal result.

**Table 2** shows that LCWG-DETR achieves an mAP of 98.24%, an mmAP of 94.18%, and a recall of 95.96% on the JXDF dataset, outperforming all comparison methods across the three accuracy metrics. Compared with the baseline RT-DETR, LCWG-DETR improves mAP, mmAP, and recall by 5.07, 5.44, and 4.48 percentage points, respectively. At the same time, its inference speed increases by 6.74 FPS, while the parameter count and computational cost are reduced by 0.35 M and 3.29 GFLOPs, respectively. These results indicate that the proposed modifications improve detection accuracy without introducing additional computational burden. Compared with YOLOv12l, LCWG-DETR achieves a 2.36 percentage point gain in mAP, uses 6.70 M fewer parameters, and increases the inference speed by 17.16 FPS, demonstrating a more favorable balance between accuracy and efficiency. Compared with the lightweight RT-DETR-EVD, LCWG-DETR improves mAP by 2.10 percentage points with an increase of only 5.51 M parameters. Although its inference speed is 15.72 FPS lower, it still reaches 92.45 FPS, which is sufficient for real time detection. LCWG-DETR also outperforms DINO and TOOD in detection accuracy while requiring fewer computational resources. Specifically, compared with DINO, it improves mAP by 1.92 percentage points and reduces the parameter count by 2.50 M. Compared with TOOD, it improves mAP by 4.00 percentage points, reduces the parameter count by 12.29 M, and lowers the computational cost by 68.0%. The performance gains result from the complementary roles of the proposed components. LGFE-ResNet18 preserves high frequency details of small lesions through local deviation texture modeling and frequency domain structural encoding at the feature extraction stage. CAFF progressively integrates shallow spatial details with deep semantic information through adaptive cross scale fusion, while WDAM guides the network toward lesion boundaries and regions with abrupt texture variations through directional modulation. GCD further improves localization by modeling bounding boxes as Gaussian distributions and providing effective regression gradients for extremely small lesions. Together, these components enable LCWG-DETR to maintain high detection accuracy while controlling model complexity and inference cost, making it well suited for the detection of small and occluded citrus disease targets in complex orchard environments.

On the OFDD dataset, LCWG-DETR also achieves the best overall detection performance, with an mAP of 96.28%, an mmAP of 82.74%, and a recall of 94.58%. Compared with the baseline RT-DETR, LCWG-DETR improves these metrics by 3.41, 5.48, and 4.00 percentage points, respectively, demonstrating that the proposed method maintains strong performance across different datasets. Compared with YOLOv12l, LCWG-DETR increases mAP by 3.07 percentage points. Relative to DINO, it improves mAP by 2.40 percentage points while using 2.50 M fewer parameters and achieving an inference speed that is 19.21 FPS higher. Compared with RT-DETR-EVD, LCWG-DETR improves mAP by 1.43 percentage points. Although its inference speed is 15.72 FPS lower, it still reaches 92.45 FPS, indicating strong real time processing capability. The images in OFDD are collected from public data sources and differ from JXDF in acquisition conditions, annotation characteristics, and the visual appearance of disease symptoms. Despite these differences, LCWG-DETR maintains consistent performance gains on OFDD. This result suggests that the ability of LGFE-ResNet18 to capture subtle texture abnormalities is not restricted to a single data distribution, supporting its transferability across datasets. The improvements introduced by CAFF and WDAM are relatively smaller on OFDD, which may be related to the lower degree of occlusion in this dataset. Nevertheless, their contribution to the Scab category indicates that these modules remain effective in capturing discriminative texture and directional information. Overall, the results on JXDF and OFDD demonstrate that LCWG-DETR achieves a favorable balance among detection accuracy, model efficiency, and real time performance. The proposed method is therefore well suited to challenging citrus disease detection scenarios involving occlusion, small lesions, and complex background interference, providing reliable visual perception support for citrus harvesting robots.

To visually compare the detection performance and disease recognition accuracy of different algorithms under complex orchard conditions, **Figure 9** presents results for four representative scenes from each of the JXDF and OFDD datasets. Columns 1 to 4 correspond to occlusion, dense disease distribution, backlighting interference, and complex backgrounds in JXDF, respectively. Columns 5 to 8 show complex backgrounds, visually similar disease appearances, densely distributed diseased fruits, and severe occlusion in OFDD, respectively. In the occlusion scene from JXDF, Faster R-CNN and YOLOv11l produce inaccurate localization results. The prediction of Faster R-CNN shifts toward a visible but nondiseased region, whereas YOLOv11l is influenced by the background dominated visible area. RT-DETR and YOLOv8m fail to detect the occluded target. In contrast, YOLOv12l, RT-DETR-EVD, DINO, and LCWG-DETR correctly identify the disease region, with the bounding box generated by LCWG-DETR showing the closest agreement with the actual lesion area. In the dense disease scene, Faster R-CNN and YOLOv8m produce false detections. Faster R-CNN generates duplicate predictions among overlapping candidate boxes, while YOLOv8m misclassifies adjacent disease regions because of insufficient separation between neighboring targets. RT-DETR and TOOD miss several lesions and fail to cover all disease regions. YOLOv11l, YOLOv12l, RT-DETR-EVD, DINO, and LCWG-DETR correctly detect all targets, among which LCWG-DETR provides the most accurate correspondence between the predicted boxes and the boundaries of individual lesions. Under backlighting interference, Faster R-CNN, YOLOv8m, YOLOv11l, RT-DETR, and DINO produce false positives by misclassifying regions with abrupt illumination changes as disease symptoms. YOLOv12l, RT-DETR-EVD, TOOD, and LCWG-DETR correctly identify the targets. LCWG-DETR provides the most accurate localization and shows stronger resistance to uneven illumination. In the complex background scene from JXDF, Faster R-CNN and YOLOv8m produce evident false detections, whereas RT-DETR and DINO miss some lesions. YOLOv11l, YOLOv12l, RT-DETR-EVD, and LCWG-DETR correctly detect all targets. Among them, the predicted boxes of LCWG-DETR show the greatest overlap with the actual lesion contours, indicating more accurate localization under complex background interference.

In the complex background scene from OFDD, Faster R-CNN generates overlapping detection boxes, whereas the remaining methods successfully detect the target. Among them, LCWG-DETR provides the most accurate localization. In the scene containing visually similar appearances, where black spot symptoms resemble healthy peel, Faster R-CNN and YOLOv8m produce false detections. In contrast, YOLOv12l, RT-DETR, RT-DETR-EVD, DINO, TOOD, and LCWG-DETR correctly classify the target, with LCWG-DETR achieving the highest confidence score of 97%. In the densely distributed diseased fruit scene, Faster R-CNN, YOLOv8m, DINO, and TOOD miss overlapping targets, while RT-DETR produces an erroneous overlapping prediction. YOLOv11l, YOLOv12l, RT-DETR-EVD, and LCWG-DETR correctly detect all disease regions, with LCWG-DETR providing the most accurate bounding box localization. Under severe occlusion, Faster R-CNN, YOLOv8m, YOLOv11l, and RT-DETR-EVD generate redundant detections, whereas RT-DETR and DINO miss some targets. Only YOLOv12l, TOOD, and LCWG-DETR correctly identify all disease regions, and LCWG-DETR retains the highest localization accuracy under extreme occlusion. Overall, the two stage structure of Faster R-CNN is susceptible to localization deviations and duplicate detections in occluded and dense scenes because candidate generation by the region proposal network may be restricted, while region pooling can introduce background interference. Although YOLOv8m and YOLOv11l employ cross level feature fusion and improved regression strategies, they remain prone to false or missed detections when targets are occluded, visually similar, or densely distributed. The global attention mechanism of RT-DETR can be dominated by nondisease regions under backlighting and complex backgrounds, thereby weakening its response to subtle lesion signals. DINO and TOOD also exhibit missed or false detections under specific interference conditions, indicating limited robustness to abrupt illumination changes and dense targets. In comparison, LCWG-DETR strengthens weak lesion features through the texture modeling and frequency domain encoding of LGFE-ResNet18. CAFF suppresses background noise and preserves lesion contours through adaptive cross scale feature fusion. WDAM enhances responses to directional texture variations while reducing nondirectional background activation. GCD further provides stronger gradient guidance for localization deviations of small targets through Gaussian distribution based bounding box modeling. The four proposed components work synergistically, enabling the model to maintain stable discriminative performance under complex conditions involving occlusion, dense target distributions, and varying illumination. This provides a practical and reliable technical solution for the visual perception system of citrus harvesting robots.

## 5 Conclusion

To address the low detection accuracy and missed detections caused by foliage occlusion, small lesions, and high interclass similarity in complex citrus orchard environments, this study proposes LCWG-DETR, a visual perception method for citrus harvesting robots. Based on RT-DETR, LCWG-DETR introduces LGFE-ResNet18, which combines local deviation guided texture modeling with global structural encoding in the Fourier frequency domain to compensate for the attenuation of high frequency information from small lesions during stacked convolution. A cross scale adaptive feature fusion module, termed CAFF, is further developed to strengthen features in occluded regions and incorporate shallow spatial details through progressive fusion and adaptive weighting. This design reduces the detail loss and noise coupling caused by upsampling in CCFM. In addition, a wavelet based directional attention module, termed WDAM, uses Haar wavelet decomposition to guide directional modulation, enabling the network to focus more accurately on lesion boundaries and regions with abrupt texture variations while reducing the dilution of weak disease responses by healthy background regions. Finally, Gaussian Combined Distance, termed GCD, is introduced as the bounding box regression loss. By modeling bounding boxes as two dimensional Gaussian distributions and jointly considering center distance and covariance information, GCD provides stronger regression gradients for extremely small lesions and alleviates gradient attenuation and scale sensitivity during small target localization. Experimental results on the JXDF and OFDD datasets show that LCWG-DETR achieves mAP values of 98.24% and 96.28%, respectively, representing improvements of 5.07% and 3.41% over the baseline RT-DETR. The proposed model reduces the parameter count by 0.35 M and reaches an inference speed of 92.45 FPS, achieving a favorable balance among detection accuracy, model complexity, and real time performance. The ablation experiments confirm the contribution of each proposed component, while the comparative results demonstrate stable detection performance and strong generalization in representative scenes involving occlusion, dense disease distributions, backlighting, and complex backgrounds. These results indicate that LCWG-DETR can provide reliable visual perception support for citrus harvesting robots. At present, the fruit detection module has been implemented and experimentally validated. Future work will focus on integrating the LCWG-DETR detector, three dimensional coordinate estimation module, and path planning algorithm into a real citrus harvesting robot platform, followed by comprehensive deployment adaptation and system level validation. The detection model will be benchmarked on embedded platforms such as NVIDIA Jetson Orin to evaluate inference speed, power consumption, and operational stability under practical deployment conditions. Model compression strategies, including TensorRT based quantized inference, structured pruning, and knowledge distillation, will also be investigated to further reduce computational latency and memory usage while preserving detection accuracy. For the complete detection, localization, and planning pipeline, parallel execution mechanisms among image acquisition, model inference, three dimensional coordinate estimation, and manipulator motion planning will be explored to reduce end to end latency. At the three dimensional localization stage, future work will further optimize subpixel centroid localization and distortion compensation to improve the robustness of spatial coordinate estimation under illumination variation, occlusion, and weak texture conditions in unstructured orchards. At the path planning stage, real time obstacle avoidance and multi target harvesting optimization will be investigated to enable the manipulator to approach target fruits efficiently and safely in complex orchard environments. These efforts will progressively establish a complete technical pipeline from algorithm validation to hardware deployment and provide practical engineering support for the integration of LCWG-DETR and the associated visual localization modules into intelligent citrus harvesting robots.

## Data Availability

The datasets presented in this study can be found in online repositories. The names of the repository/repositories and accession number(s) can be found in the article/supplementary material.
